# Is age‐related increase of chromosome segregation errors in mammalian oocytes caused by cohesin deterioration?

**DOI:** 10.1002/rmb2.12299

**Published:** 2019-09-12

**Authors:** Jibak Lee

**Affiliations:** ^1^ Laboratory of Developmental Biotechnology Graduate School of Agricultural Science Kobe University Kobe Japan

**Keywords:** age‐related aneuploidy, chromosome segregation, cohesion, oocyte, sister chromatid cohesion

## Abstract

**Background:**

Mammalian oocytes initiate meiosis in fetal ovary and are arrested at dictyate stage in prophase I for a long period. It is known that incidence of chromosome segregation errors in oocytes increases with advancing age, but the molecular mechanism underlying this phenomenon has not been clarified.

**Methods:**

Cohesin, a multi‐subunit protein complex, mediates sister chromatid cohesion in both mitosis and meiosis. In this review, molecular basis of meiotic chromosome cohesion and segregation is summarized. Further, the relationship between chromosome segregation errors and cohesin deterioration in aged oocytes is discussed.

**Results:**

Recent studies show that chromosome‐associated cohesin decreases in an age‐dependent manner in mouse oocytes. Furthermore, conditional knockout or activation of cohesin in oocytes indicates that only the cohesin expressed before premeiotic S phase can establish and maintain sister chromatic cohesion and that cohesin does not turnover during the dictyate arrest.

**Conclusion:**

In mice, the accumulating evidence suggests that deterioration of cohesin due to the lack of turnover during dictyate arrest is one of the major causes of chromosome segregation errors in aged oocytes. However, whether the same is true in human remains elusive since even the deterioration of cohesin during dictyate arrest has not been demonstrated in human oocytes.

## 1 INTRODUCTION

Gametes (ova and spermatozoa) are produced from diploid germ cells (oocytes and spermatocytes) through a special type of cell division called meiosis. Meiosis consists of two successive nuclear divisions without intervening S phase. In the first meiotic division (meiosis I), homologous chromosomes establish the connection with their partners at prophase, forming bivalent chromosomes. Then, at the onset of anaphase I, homologous chromosomes separate from each other by the dissolution of cohesion along chromosome arm region while sister chromatids remain attached at centromere region. In the second meiotic division (meiosis II), sister chromatids separate from each other by the dissolution of the remaining centromeric cohesion.^1^, ^2^

**Figure 1:**
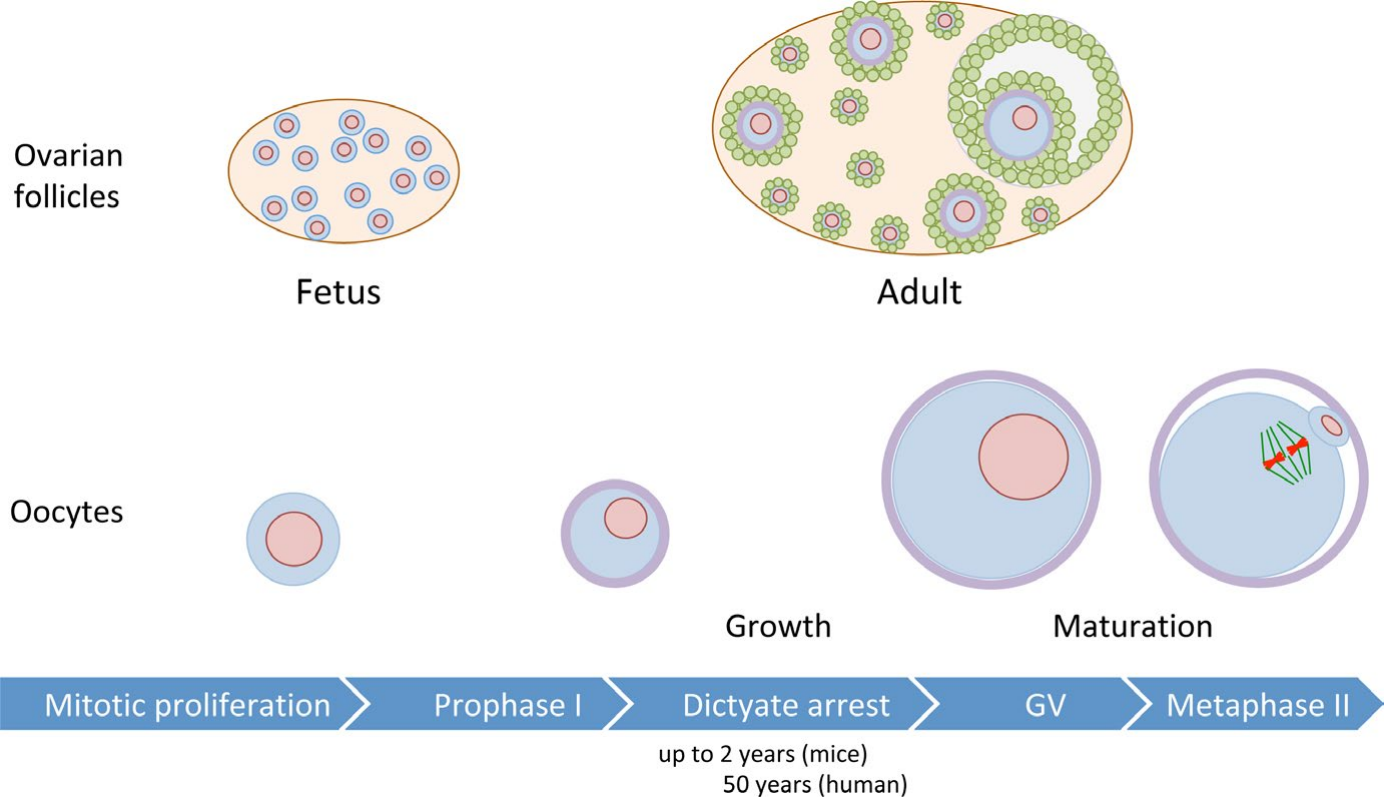
Oogenesis in mammals. Mammalian oocytes initiate meiosis following proliferation by mitotic cell division in fetal ovary. The oocytes conduct synapsis and recombination of homologous chromosomes, progress to diplotene stage at which the first meiotic arrest is imposed. During this arrest, chromatin is decondensed and diffusely distributed unlike male meiosis, and this female meiosis‐specific stage is also called dictyate stage. After birth, some of oocytes start to grow with the development of the surrounding follicles in an estrous cycle dependent manner. Prior to ovulation, oocytes conduct maturation by progressing from GV stage to metaphase II, at which fertilization occurs

Meiosis is sexually dimorphic in mammalian gametogenesis.^2^, ^3^, ^4^ In males, meiosis initiates after puberty and proceeds continuously to the end of meiosis followed by gametogenic differentiation from round spermatids into spermatozoa. In the adult male gonad, meiotic cells called spermatocytes are continuously provided from mitotically dividing stem cells called spermatogonia. In contrast, in females, the number of oocytes reaches a peak at fetal stage and decreases as females get older. Female meiosis starts in the fetal stage (eg, 13.5 days post‐coitum in mice) and proceeds to the extended diplotene stage, also called dictyate stage, at which the first meiotic arrest is imposed (Figure 1). During this arrest, only a limited number of oocytes grow their volume with development of the surrounding follicle. Then, fully grown oocytes resume meiosis and are ovulated at metaphase II stage in each estrous cycle until females go into menopause. The progression from germinal vesicle (GV) stage to metaphase II stage is so‐called oocyte maturation. During oocyte maturation, oocytes conduct germinal vesicle breakdown, segregate homologous chromosomes, and emit the first polar body, and arrest again at metaphase II. During this arrest, oocytes are ovulated and fertilized with spermatozoa. The fertilized oocytes finally complete meiosis by segregating sister chromatids and emitting the second polar body. Hence, in mammalian ovary, majority of oocytes remains arrested at dictyate stage for months, years, or decades depending on species' reproductive lifespans.

The rate of aneuploidy of fertilized eggs in human has been reported to be 10%‐35% from 1990s studies and 30%‐70% in more recent studies, which is much higher than those in other species, for example, 1%‐2% in mice.^5^, ^6^ In human, the incidence of some birth defects or congenital disorders increases as the age of pregnant female increases.^5^ Although chromosome errors can occur in both male and female gametogenesis, paternal age does not contribute to age‐related increase in aneuploidy because cells having chromosome errors are mostly eliminated by cell cycle checkpoint mechanisms regardless of paternal age.^7^ Thus, the age‐related increase of aneuploidy in fertilized eggs is caused by the increase in oocyte aneuploidy. Since oocytes, unlike spermatogenesis, undergo meiosis at puberty and arrest at meiotic prophase I for long period, it has been thought that deterioration of some key molecules during the prolonged prophase I leads to chromosome segregation errors in aged oocytes. Recent studies suggest that cohesin, a glue protein complex for sister chromatid cohesion, is the most likely candidate for the key molecule.

## 2 SISTER CHROMATID COHESION MEDIATED BY COHESIN

### 2.1 Sister chromatid cohesion in somatic cells

In eukaryotes, sister chromatid cohesion must be maintained from S phase to the onset of anaphase for the accurate transmission of genome from mother cells to daughter cells. Cohesin, a multi‐subunit protein complex well conserved among eukaryotes, mediates sister chromatid cohesion.^8^, ^9^, ^10^ The cohesin complex consists of two structural maintenance of chromosome proteins, SMC1α and SMC3, and two non‐SMC proteins, either one of SA1 and SA2, and RAD21 (also called SCC1) (Table 1). Cohesin forms a tripartite ring‐like structure that is composed of V‐shaped SMC1α‐SMC3 heterodimer and RAD21 connecting the heads of the V‐shaped dimer.^11^ It is believed that cohesin binds sister chromatids by topologically embracing them in its ring‐like structure.^12^ In the mitotic cell cycle, cohesin is loaded on chromatin through SCC2 (NIPBL)‐SCC4 (MAU2) cohesin loader before DNA replication.^13^ The function to mediate sister chromatid cohesion is exerted by cohesin which has been recruited at or before DNA replication in mitotic cell cycle.^14^, ^15^ Establishment of sister chromatid cohesion by cohesin requires acetyl transferases, ESCO1 and ESCO2, which acetylates lysine residues of SMC3.^16^, ^17^ Although cohesin can bind to DNA in an ATPase‐independent manner, topological binding of cohesin to DNA requires ATP hydrolysis by SMC subunits.^12^ For the entrapment of DNA in cohesin ring, the ring must be opened. The ring opening occurs in two postulated ways: SMC1α‐SMC3 interphase or SMC3‐RAD21 interphase.^18^ The latter is known to function as “exit gate”. WAPL‐PDS5 open the SMC3‐SCC1 gate and is required for the release of cohesin from chromosomes which promotes sister chromatid resolution prior to anaphase.^19^ Sororin competes with WAPL for binding to PDS5 and antagonizes WAPL, thereby stabilizing cohesin on chromatin.^20^, ^21^ Sororin only binds to cohesin only after SMC3 is acetylated.^21^

**Table 1:** Subunits and accessary proteins of cohesin complex in mammals

	Type	Mitotic (ubiquitous)	Meiosis‐specific
Cohesin subunits	SMC	SMC1α SMC3	SMC1β
	Kleisin	RAD21	REC8 or RAD21L
	HEAT repeat	STAG1/SA1 or STAG2/SA2	STAG3/SA3
Cohesin regulators	Loading	SCC2/NIPBL SCC4/MAU2	
	Stabilization and/or removal	ESCO1, ESCO2 PDS5A, PDS5B SORORIN WAPL	

### 2.2 Sister chromatid separation at anaphase

In prophase, most of cohesins dissociate from chromosome arms by so‐called prophase pathway which is dependent on WAPL‐PDS5 as well as phosphorylation of SA subunits.^22^ Then, after nuclear envelope disassembly, condensed chromosomes start to align at spindle equator by capturing spindle microtubules at kinetochores. Sister kinetochores attach to the microtubules extended from the opposite poles of spindle, thereby establishing the bipolar attachment. When all the kinetochores establish the bipolar attachment, spindle assembly checkpoint permits sister chromatids to separate in anaphase by the activation of anaphase‐promoting complex/cyclosome (APC/C).^23^ The activation of APC/C involves the association of its activator CDC20. APC/C^CDC20^ ubiquitinates its target proteins, cyclin B and securin, thereby inducing degradation of them by proteasome.^24^, ^25^ The degradation, in turn, brings about activation of separase, which cleaves a kleisin subunit RAD21.^26^ This induces the irreversible opening of cohesin ring, which brings about sister chromatid separation.

## 3 ESTABLISHMENT OF LINKAGE BETWEEN HOMOLOGOUS CHROMOSOMES AT PROPHASE I

### 3.1 Synapsis and recombination of homologous chromosomes

For proper segregation of chromosomes in meiosis, homologous chromosomes must be linked with their partners in prophase I. Meiotic recombination (crossover recombination) contributes not only to increasing the genetic diversity in gametes but also to creating the linkage between homologous chromosomes. Recombination initiates by a programed DNA double‐strand break (DSB) with Spo11.^27^, ^28^ The DSBs are repaired through several intermediates such as extended D‐loop and double Holliday junction and finally resolved into crossovers and noncrossovers.^29^ In parallel with the recombination process, homologous chromosomes are juxtaposed and connected with each other by the assembly of the synaptonemal complex (SC).^30^ At leptotene stage, an axial element (AE) is formed on each chromosome, then the AEs of homologous chromosomes start to be connected by transverse filaments at zygotene stage. Homologous chromosomes are fully connected along the entire length by the assembly of the SC by pachytene stage. The SC assembly is required for proper formation of crossover recombination.^31^ The connection mediated by SC is only temporal from the assembly of SC at zygotene until the disassembly of the SC at diplotene stage. At later meiotic stages, the connection between homologous chromosomes is maintained by crossover recombination‐mediated linkage. Therefore, crossover formation is tightly regulated to assure accurate chromosome segregation in meiosis I. Firstly, every pair of homologous chromosomes receives at least one crossover, which is called an obligate crossover. Secondly, if multiple crossovers occur, one does not tend to be close to the others, which is so‐called crossover interference. Thirdly, when DSB formation is altered, crossover levels are maintained presumably at the expense of noncrossover.^29^, ^32^

### 3.2 Meiotic cohesins

**Figure 2:**
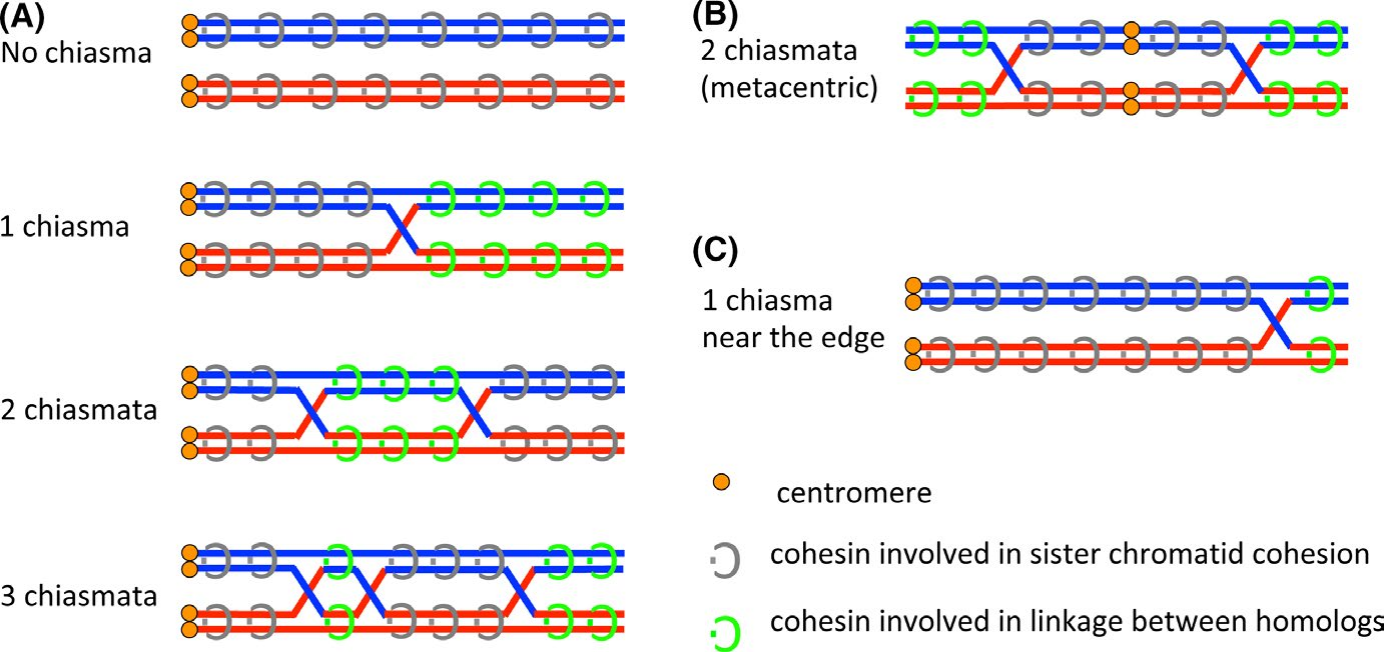
Linkage between homologous chromosomes through chiasma and cohesion. A, Pairs of homologous telocentric chromosomes are described. When no chiasma is present, all cohesins on the chromosomes mediate sister chromatid cohesion. Once a chiasma is formed, cohesins localizing on chromosome arms distal to chiasma (in relation to centromeres) become involved in linkage between homologous chromosomes. When two chiasmata are formed, only the cohesins localizing on chromosome arms between two chiasmata become responsible for the homologous chromosomes linkage. When three chiasmata are formed, the cohesin localizing both between the first and second chiasmata and distal to the third chiasma become responsible for the homologous chromosomes linkage. B, A pair of homologous metacentric chromosomes is described. When two chiasmata are formed on both sides of chromosome arms in relation to centromeres, cohesins localizing distal to chiasmata are responsible for linkage between homologous chromosomes. C, When a chiasma is formed near the edge of telocentric chromosomes, the cohesins involved in linkage between homologous chromosomes are fewer than when a chiasma is formed at the middle of chromosome arms as described in A

After the first discovery of Rec8 as a meiotic counterpart of Rad21 in yeast,^33^, ^34^ several kinds of meiosis‐specific subunit have been found in various eukaryotes.^35^ In mammals, four meiosis‐specific subunits, REC8,^36^, ^37^ RAD21L,^38^, ^39^, ^40^ SMC1β,^41^ and STAG3,^42^ have been found so far and all the meiosis‐specific subunits as well as mitotic cohesin subunits^43^, ^44^, ^45^ are localized on the synaptonemal complex (Table 1).^46^, ^47^ Genetic studies using knockout mice have revealed that all the meiosis‐specific cohesin subunits are required for the process of establishing the linkage between homologous chromosomes, such as axial element formation, synapsis, and recombination of homologous chromosome, although different phenotypes have been observed depending on the subunit.^48^, ^49^, ^50^, ^51^, ^52^, ^53^, ^54^, ^55^, ^56^, ^57^, ^58^ For example, absence of either one of REC8,^48^, ^50^ RAD21L,^53^ or SMC1β,^49^, ^52^ causes the partial defects of axial element formation, whereas absence of STAG3 abolishes axial element formation.^55^, ^56^ Furthermore, it has been suggested that RAD21L‐containing cohesin may function for the establishment of linkage between homologs whereas REC8‐containing cohesin may function for both homologous chromosome linkage and sister chromatid cohesion.^47^, ^57^, ^59^ The separation of roles by different cohesins in meiosis might be conserved among species.^60^ Once the crossover recombination between homologous chromosomes has been established, a DNA molecule of one homologous chromosome is interlinked to a DNA molecule of another homologous chromosome. As a result, crossover recombination converts the sister chromatid cohesion distal to chiasma (the position of crossover recombination) into the non‐sister chromatid cohesion between homologous chromosomes (Figure 2). Thus, the linkage between homologous chromosomes is maintained by the function of cohesin to mediate sister chromatid cohesion with the aid of crossover recombination.

## 4 CHROMOSOME SEPARATION DURING MEIOSIS

In order to segregate homologous chromosomes in meiosis I, sister kinetochores on a univalent chromosome must be attached to the spindle microtubules emanated from the same spindle pole. This is so‐called monopolar attachment requiring centromeric cohesion as well as meiosis‐specific kinetochore protein MEIKIN.^35^, ^61^ When all the kinetochores establish the monopolar attachment and bivalent chromosomes align on the spindle equator, APC/C is activated by the binding of CDC20.^62^ The APC/C^CDC20^ induces the degradation of cyclin B and securin by ubiquitinating them, which in turn activates separase.^63^, ^64^, ^65^, ^66^ The activated separase cleaves REC8,^67^, ^68^, ^69^ thereby removing cohesin from chromosome arms while centromeric cohesin is protected by shugoshin, SGO2 in mammals.^70^, ^71^ Since the linkage between homologous chromosomes is maintained by sister chromatid cohesion distal to a chiasma, cohesin release from chromosome arm region induces homologous chromosome separation.

## 5 CHROMOSOME SEGREGATION ERRORS IN MEIOSIS

**Figure 3:**
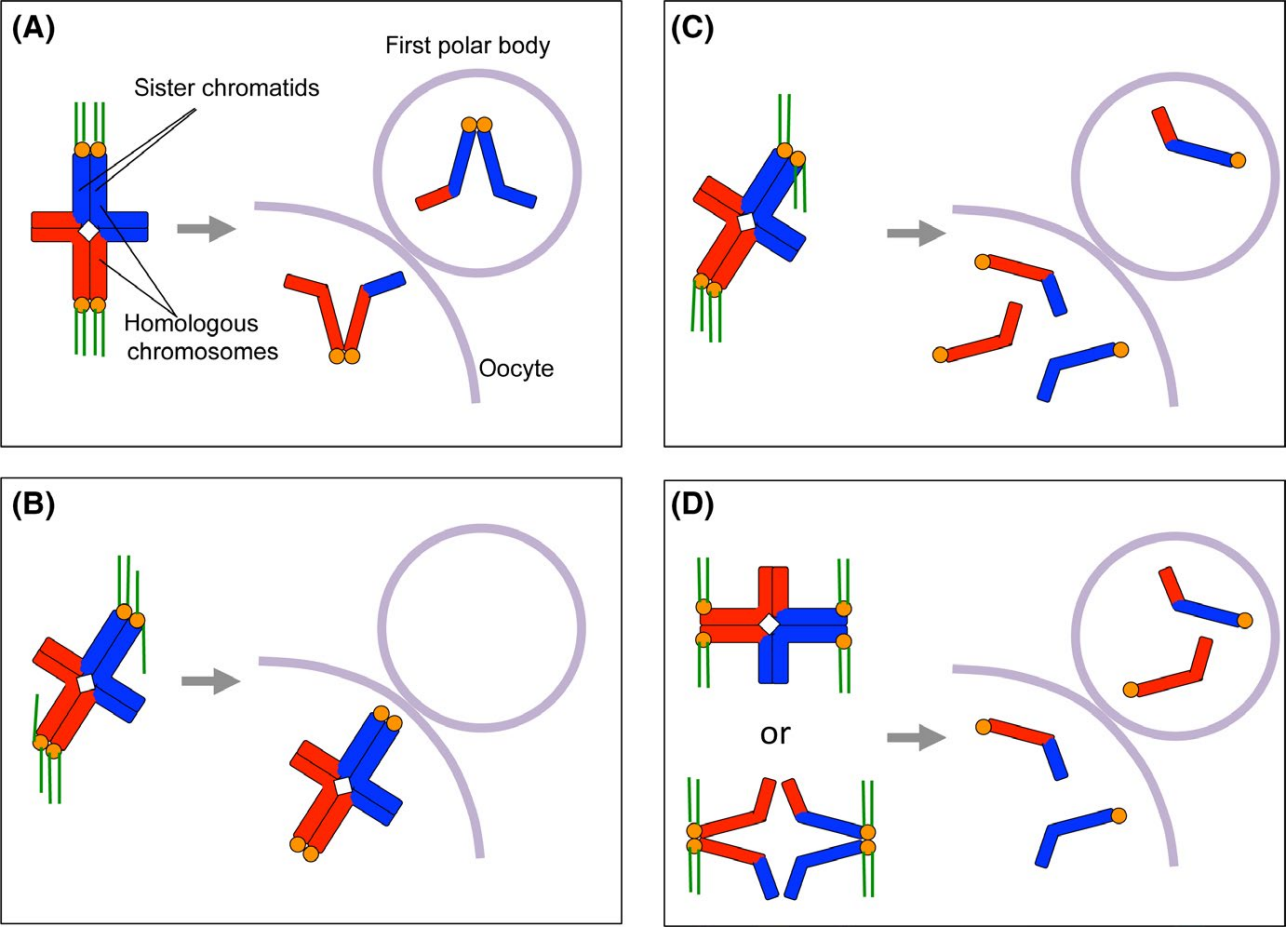
Chromosome segregation and its possible errors in meiosis I. A, In meiosis I, two sister kinetochores on a univalent establish the monopolar attachment to the spindle microtubules so that homologous chromosomes can separate in anaphase I by the dissolution of chromatid arm cohesion. B, When two sister kinetochores on a univalent cannot establish the monopolar attachment, homologs cannot separate in anaphase I, resulting in nondisjunction of homologous chromosomes. C, When two sister kinetochores on a univalent attach to microtubules in a half‐inverted manner instead of monopolar attachment, some of sister chromatids separate precociously in anaphase I. As a result, the secondary oocyte receives an extra number or lesser number of chromatids. D, When centromeric cohesion is precociously lost, sister kinetochores no longer behave as a single unit, resulting in the establishment of bipolar attachment. When chiasma is dissolved by premature loss of arm cohesion, sister kinetochores tend to establish bipolar attachment. In both case, correct number of chromatids might be distributed into the secondary oocyte and the first polar body after anaphase I. However, in meiosis II, chromosome separation does not occur accurately due to the lack of sister chromatid cohesion

Chromosome segregation errors in meiosis occur in some different ways, namely nondisjunction, premature separation of sister chromatids, and reverse segregation (Figure 3). Nondisjunction occurs when chromosome segregation fails in meiosis I or meiosis II probably due to the faulty attachment of chromosomes to spindle microtubules such as merotelic attachment instead of amphitelic attachment.^72^ However, recent studies examining aneuploidy in oocytes by counting the number of chromosomes in the first polar body suggest that errors caused by premature separation of sister chromatids are more common than those caused by nondisjunction in meiosis I.^72^, ^73^, ^74^ Premature sister chromatid cohesion in meiosis I involves untimely dissociation of cohesin from chromosomes or deterioration of cohesin. Reverse segregation occurs when sister chromatids, but not homologous chromosomes, segregate in meiosis I.^72^ As a result, a correct number of chromosomes are distributed to the secondary oocyte and the first polar body. But, in meiosis II, chromosomes cannot segregate correctly due to the lack of centromeric cohesion, which should be maintained in normal metaphase II oocytes until fertilization. Although it has been considered that differences in cell size, chromosome geometry, spindle assembly, and cell cycle control between oocytes and somatic cells render oocytes so prone to segregation errors,^75^, ^76^, ^77^ I limit the discussion to chromosome cohesion in this review.

## 6 COHESIN DETERIORATION IN AGED OOCYTES DUE TO THE ABSENCE OF TURNOVER AT PROPHASE I ARREST

### 6.1 Decrease of chromosome‐associated cohesin in aged oocytes

**Figure 4:**
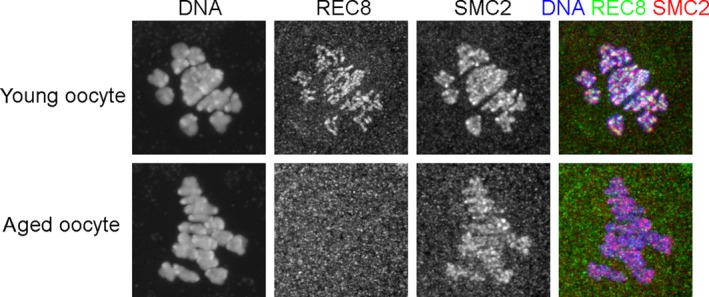
Chromosome‐associated cohesin is decreased in mouse oocytes with advancing age. GV stage oocytes derived from 3‐week‐old or 13‐month‐old mice were cultured for 6 hr in vitro. After culture, the oocytes progressed to metaphase I and were fixed and immunofluorescently labeled with anti‐REC8 antibody and anti‐SMC2 antibody. DNA was counterstained with DAPI. SMC2, one of the subunits of condensin, are detected on chromosomes in both young and aged oocytes. In contrast, REC8 is hardly detected on chromosomes in the aged oocytes although it is clearly detectable in young oocytes

In mitotically proliferating cells, cohesin that associates with DNA at or prior to S phase can mediate sister chromatid cohesion. If this is applicable to meiosis, only the cohesin expressed in the oocytes in fetal ovary can contribute to meiotic sister chromatid cohesion since mammalian oocytes undergo premeiotic DNA replication at fetal stage. Considering that mammalian oocytes arrest at dictyate stage for a long time up to decades in some species, one can imagine that cohesin might decrease during the arrest. In fact, as has been reported previously,^78^, ^79^, ^80^ REC8 is hardly detected on bivalent chromosomes at metaphase I in aged oocytes from 13‐month‐old mice whereas it is clearly observed in young oocytes from 3‐week‐old mice (Figure 4). Unlike cohesin, another chromosome‐associated SMC protein complex called condensin^81^ is detectable in both young and aged oocytes (Figure 4). Since substantial amount of condensin is recruited to chromosomes after nuclear envelope disassembly and functions in chromosome condensation and segregation in both mitosis and meiosis,^82^, ^83^, ^84^ prolonged arrest at dictyate stage may not affect the amount of chromosome‐associated condensin in the aged oocytes. It has also been reported that chromosome‐associated REC8 decreases gradually from 3 months old to 9 months old whereas thereafter REC8 level cannot be measured accurately due to the limitation of the detection or quantification ability.^79^ From the data, half‐life of the chromosome‐associated REC8 seems to be about 2 months during the period. Also, in human, it has been reported that cohesin decreases in aged oocytes, but the amount of reduction is much smaller than that reported in mouse oocytes: More than half amount of cohesin subunits, REC8 and SMC1β, are still retained in the oocytes derived from women aged 40 years and over compared with those aged around 20 years.^85^ In the human study, the signal intensity might be affected by chromatin‐unbound cohesin in the nucleus, since they estimated the relative cohesin levels by examining the signal intensity of cohesin within nucleus at dictyate stage in contrast to the study in mice using metaphase I‐stage oocytes. Another study examining the cohesin on chromosome spreads from human oocytes has reported that there are no evident differences in the intensity and distribution among oocytes derived from women aged between 18 and 34 years old.^86^ In summary, it is evident that chromosome‐associated cohesin decreases in oocytes as females get older in mice, but absolute evidence concerning this issue has not been obtained in human.

### 6.2 No cohesin turnover during the dictyate arrest in mouse oocytes

In general, the amount of proteins is determined by rate of synthesis and degradation. Synthesis of cohesin subunits seems to continue in the oocytes after birth because *Rec8* mRNA was detected in the ovarian oocytes in adult mice.^87^ Indeed, immunoblot analysis reveals that the total amount of the proteins is not different between young and aged oocytes.^79^ So, it is thought that decrease of chromosome‐associated cohesin would be caused by deterioration of cohesin without turnover during the meiotic arrest. To test this hypothesis, conditional knockout or activation strategies have been utilized in mice. A study using REC8^TEV/TEV^ mice, which expresses artificially‐cleavable REC8 by TEV protease, show that injection of TEV protease into metaphase I oocytes induces the premature sister chromatid separation. But, in the oocytes derived from the REC8^TEV/TEV^ mice, the expression of ectopic REC8‐Myc activated by *Sox9‐Cre* transgene prior to premeiotic DNA replication prevents the TEV protease‐mediated premature sister chromatid separation while the expression of REC8‐Myc activated by *Zp3‐Cre* transgene during dictyate arrest at the onset of oocyte growth does not rescue the premature sister chromatid separation.^88^ This suggests that REC8 can establish and maintain sister chromatid cohesion when it is expressed only prior to premeiotic S phase but not when expressed in later meiotic stages. Furthermore, a recent study using drug‐inducible Cre system also supports this notion.^89^ In addition, inactivation of *Smc1β* gene shortly after birth at dictyate arrest in oocytes does not affect chiasma positions and sister chromatid cohesion^90^ whereas conventional knockout of *Smc1β* does.^49^ These studies strongly suggest that only the meiotic cohesin expressed before or during premeiotic S phase can establish and maintain sister chromatid cohesion and that there is little or no cohesin turnover during the meiotic arrest at prophase I and thereafter.

### 6.3 Types of cohesin whose deterioration is potentially involved in age‐related increase of aneuploidy

Supposing that decrease in cohesin causes the age‐related increase of aneuploidy, what types of cohesin relevant to this issue? Each of cohesin subunits except SMC3 has multiple variants including meiosis‐specific one (Table 1). Among three kleisin subunits, RAD21L is expressed mainly in the early stages of prophase I and hardly detected at later meiotic stages.^38^, ^39^ Thus, it is thought that RAD21L cannot maintain the sister chromatid cohesion during dictyate arrest. RAD21 does not contribute to sister chromatid cohesion in meiosis since it has been demonstrated that RAD21 cleavage by TEV protease triggers sister chromatid separation in the first embryonic mitosis but not homologous chromosome separation during meiosis.^88^ Therefore, neither RAD21L nor RAD21 are involved in the age‐related increase of aneuploidy. In contrast, REC8 localizes to the connection sites between chromatids until metaphase II^66^ and is proved to induce precocious sister chromatid separation in meiosis I when it is cleaved by TEV system.^88^ Moreover, chromosome‐associated REC8 is indeed decreased in aged oocytes^79^, ^80^ (Figure 4). So, among three kleisin subunits, REC8 is the only subunit whose deterioration would be able to cause an increase of chromosome segregation errors in aged oocytes. Among three SA subunits, only STAG3 has been shown to remain at the connection sites of chromatids at least until metaphase I.^42^ Furthermore, co‐expression of STAG3 with REC8 in somatic cells enables REC8 to enter the nucleus and functionally replace its mitotic counterpart RAD21 and can rescue precocious sister chromatid separation induced by overexpression of hyperactive separase or knockdown of Sgo1 in somatic cells, suggesting that REC8‐STAG3 cohesin can mediate sister chromatid cohesion.^91^ So, STAG3 is the most likely candidate. Among two SMC1 subunits, SMC1β but not SMC1α localizes on chromosomes after diplotene^41^ and is associated with REC8 in testis extracts of wild‐type mice.^37^ However, since SMC1α can substitute for many SMC1β functions,^92^ SMC1α might mediate sister chromatid cohesion after diplotene if SMC1β is reduced during dictyate arrest. In summary, REC8‐STAG3‐SMC3‐SMC1β (also SMC1α if present after diplotene stage) is likely to be responsible for the maintenance of sister chromatid cohesion at dictyate arrest and thereafter. Thus, its decrease possibly leads to age‐related increase of chromosome segregation errors in oocytes.

### 6.4 Possible mechanism to reduce chromosome‐associated cohesin in dictyate‐arrested oocytes

How is chromosome‐associated cohesin decreased during meiotic arrest of oocytes? Recent studies have reported the expression of cohesin regulators in meiocytes. NIPBL, a cohesin loading factor, is associated with the AEs of the SC from leptotene to diplotene stage but hardly detected at dictyate stage in mouse oocytes.^93^, ^94^ These reports imply that newly synthesized cohesin may be loaded to meiotic chromosomes until diplotene stage but not during dictyate arrest, supporting the hypothesis of no cohesin turnover during dictyate arrest. Human *Wapl* mRNA and mouse WAPL protein, a removing factor of cohesin, is highly expressed in testis and is localized on AEs the SC in zygotene and pachytene spermatocytes^95^ and also localized on the SC in pachytene oocytes.^96^ NIMA‐like kinase‐1 (NEK1) phosphorylates PP1, leading to the dephosphorylation of WAPL, which in turn results in its retention on chromosome cores to promote loss of cohesion at the end of prophase I.^97^ These studies suggest that WAPL functions in removal of cohesin also in meiosis. Sororin, a stabilizing factor of cohesin, is localized to the central region of the SC interestingly in a synapsis‐dependent manner, but a meiotic cohesin‐independent manner at zygotene and pachytene stages, then accumulate at centromere by late prophase I, and remains there until anaphase II in mouse spermatocytes.^98^, ^99^ PDS5B, a cohesin regulator associating alternatively with WAPL or sororin, starts to be detected on the AEs at zygotene stage, culminates at pachytene stage, and is diminished at diplotene stage in mouse spermatocytes.^100^ Immunoprecipitation analyses reveal that PDS5B is associated with SYCP2 and cohesin subunits such as SMC1β and REC8.^100^ Furthermore, ectopic expression of meiosis‐specific cohesin subunits reveals that REC8‐STAG3 cohesin physically interacts with PDS5, WAPL, and sororin and that REC8‐STAG3 cohesin is shown to be susceptible to WAPL‐dependent removal and sororin‐mediated protection.^91^ Overall, WAPL, sororin, and PDS5B are involved in regulation of meiotic cohesin. Thus, untimely activation of WAPL and/or inactivation of sororin might cause the reduction of chromosome‐associated cohesin during dictyate arrest in oocytes I deleted the above sentences according to the suggestion of the reviewer. Since SGO2, a centromeric cohesin protector, is reduced in aged oocytes, the reduction may amplify the cohesin loss during dictyate arrest.^80^

Little is known about phosphorylation of SA subunits in meiosis except that RAD21L‐associated STAG3 (SA3) is detected as phosphorylated form.^38^ Whether the phosphorylation of STAG3 associating with REC8 occurs during dictyate arrest should be tested in future studies. Besides above regulations, cohesin can be removed from chromosomes by separase‐dependent cleavage of a kleisin subunit. However, considering the cell cycle stage of meiotic arrest of oocytes, separase‐dependent pathway is not likely unless precocious activation of separase occurs. This is supported by the report that spindle checkpoint function is not impaired in aged oocytes.^80^

## 7 IS AGE‐RELATED INCREASE IN CHROMOSOME SEGREGATION ERRORS CAUSED BY COHESIN DETERIORATION?

### 7.1 Mouse oocytes

It is evident that cohesin deterioration occurs in aged mouse oocytes due to the lack of turnover during dictyate arrest. It is also shown experimentally that total loss of cohesin in metaphase I leads to precocious sister chromatid separation.^88^ Thus, it seems reasonable to conclude that deterioration of cohesin is one of the major causes for the increase of chromosome segregation errors in aged mouse oocytes. Then, is there any room for argument on this point? In this regard, it must be noted that there is no evidence to show the exact correlation between cohesin decrease and increase of chromosome segregation errors during aging of oocytes. Chromosome‐associated REC8 in mouse oocytes gradually decreases and reaches the bottom level at 9 months old while chromosome segregation errors increase abruptly at 15‐month‐old.^79^ So, there is a discrepancy in the timing. This discrepancy may be accounted for by the hypothesis that there is a threshold of cohesin level below which sister chromatid cohesion cannot be maintained and that cohesin continues to decrease to the threshold level below the detection limit of the current analysis. But, the hypothesis seems unsound in that a result may be able to be interpreted in two contrary ways. For example, the REC8 staining in an aged oocyte in Figure 4 would be regarded as no or little cohesin when you want to insist the age‐dependent deterioration of cohesin, whereas it would be regarded as the presence of a very small amount of cohesin under detection limit when you want to insist the maintenance of sister chromatid cohesion by the invisible cohesin. Furthermore, there are some issues to consider about threshold; for example, which region of chromosome cohesin dissociates from, and whether functional loss of cohesin occurs. As shown in Figure 2, to maintain the link between homologous chromosomes, the cohesin localizing distal to chiasma should remain functional. So, even if net amount of chromosome‐associated cohesin might remain at a sufficient level in oocytes, the loss of the cohesin functioning for homologous chromosome link (green‐colored cohesin in Figure 2) would evoke chromosome segregation error. We need to know the local threshold level of meiotic cohesin to show a clear correlation between cohesin decrease and increase of chromosome segregation errors during aging of oocytes.

### 7.2 Human oocytes

As mentioned above, there is no conclusive evidence showing that chromosome‐associated cohesin is decreased in human aged oocytes. Moreover, whether turnover of cohesin occurs or not during dictyate arrest cannot be tested experimentally in human oocytes. Therefore, there are not much data to decide this issue. Comparing human oocytes with mouse oocytes, the timing of increase of chromosome segregation errors is different: The incidence of chromosome segregation errors in oocytes increases over 1 year old in mice while it does over 35 years old in human. What makes the timing so different between these two species is unknown. If cohesin does not turnover during dictyate arrest in human oocytes as seen in mice, meiotic cohesin in human oocytes should be extremely long‐lived proteins. In general, proteins turnover within a few days but a few such as histones, nuclear pore complex proteins, show remarkable stability: The half‐life of histone is ~200 days and that of scaffold proteins of nuclear pore is more than 1 year.^101^, ^102^ In addition, the half‐life of histones within cells is estimated to be 117 days in liver and 223 days in brain.^101^ Thus, half‐life of a protein seems to be dependent on both its property and the surrounding environment. In this context, meiotic cohesin should have its own property of longevity, and human oocytes may provide much better condition for the preservation of long‐life proteins than mouse oocytes if the hypothesis of no cohesin turnover is applied to human oocytes.

## 8 CONCLUSION

In brief, accumulating evidence in mouse oocytes suggests that deterioration of cohesin is one of the major causes for the age‐related increase in chromosome segregation errors, but we still cannot rule out the possibility that deterioration of molecules other than cohesin or mechanisms might trigger the final switch to chromosome segregation errors in aged oocytes even in mice. CENP‐A, a centromere‐specific histone H3 variant, is shown to persist more than 1 year without detectable turnover in mouse oocytes.^103^ Therefore, reduction of such long‐lived proteins in combination with cohesin deterioration during oocyte aging might give a deleterious effect on chromosome segregation. In *Drosophila* oocytes, loading of newly synthesized cohesin by Nipped‐B and establishment of new cohesive linkage by Eco during prophase I after premeiotic S phase is required for maintaining cohesion although the rejuvenation might be needed at earlier stages than the stage at which mammalian oocytes are arrested.^104^ Further studies examining the cohesin turnover in other species are needed to understand the underlying mechanism of age‐related aneuploidy in human and other mammalian oocytes.

## DISCLOSURES

Conflict of interest: Jibak Lee declares that he has no conflict of interest.

Human Rights: This article does not contain any studies with human subjects performed by any of the authors.

Animal studies: This study was approved by the Institutional Animal Care and Use Committee (Permission number: R1‐27‐03‐01) and carried out according to the Kobe University Animal Experimentation Regulations.
